# Transcriptome and Coexpression Network Analyses Provide Insights into the Resistance of Chinese Cabbage During Different Stages of *Plasmodiophora brassicae* Infection

**DOI:** 10.3390/plants14142105

**Published:** 2025-07-08

**Authors:** Huishan Liu, Lili Wang, Guozheng Wang, Haidong Wu, Xin Wang

**Affiliations:** Vegetable Research Institute, Liaoning Academy of Agricultural Sciences, Shenhe District, 84 Dongling Road, Shenyang 110161, China; liuhuishan1993@163.com (H.L.); wanglili_81@163.com (L.W.); wanggzcow@163.com (G.W.)

**Keywords:** clubroot, Chinese cabbage, weighted gene coexpression network analysis, regulatory network

## Abstract

Clubroot is a destructive soilborne disease caused by *Plasmodiophora brassicae* that threatens the production of Chinese cabbage. The molecular mechanisms underlying the resistance of Chinese cabbage to clubroot remains unclear, making the identification and analysis of resistance genes crucial for developing resistant varieties. Comparative transcriptome analysis of roots from the resistant line “JJ S5-1” and the susceptible line “SYY10-1” revealed significant differences in gene expression profiles at various stages after inoculation. Weighted gene coexpression network analysis revealed midnight blue and green modules as substantially associated with disease response, with each showing positive regulatory patterns. Several defense-related genes and transcription factors important for resistance to *Plasmodiophora brassicae* were identified, including disease resistance proteins, PR1, PBS1, and TGA, and WRKY transcription factors, most of which were upregulated following inoculation. Key genes associated with trait-related expression patterns were analyzed and a working model was proposed to explain the mechanism of clubroot disease resistance to *Plasmodiophora brassicae* infection in Chinese cabbage. These findings offer a valuable resource for further investigation of the immune response in the resistance of “JJ S5-1” to clubroot disease.

## 1. Introduction

Clubroot is caused by *Plasmodiophora brassicae*, a soilborne protist and obligate biotrophic parasite that infects the roots of cruciferous crops, inducing root swelling and galls that reduce crop quality and yield [1]. Additionally, *P. brassicae* produces large quantities of resting spores that can remain viable in the soil for 8–12 years or longer [2]. This disease causes substantial crop damage in more than 60 countries, leading to global yield losses of 10–15% [3]. In recent years, the incidence of clubroot disease has steadily increased in southeastern, northeastern, and central China [4]. Consequently, clubroot remains difficult to control and continues to garner widespread attention.

Plants are continually exposed to pathogens, including bacteria, viruses, and fungi, throughout their growth and development [5]. In response, plants have evolved signal transduction pathways that enable interactions with pathogens, leading to enhanced disease resistance [6]. The plant immune system comprises two primary types: pathogen/microbe-associated molecular pattern (PAMP/MAMP)-triggered immunity (PTI) and effector-triggered immunity (ETI) [7]. PTI acts as the first barrier, recognizing PAMPs via pattern-recognition receptors on the cell surface, which activates the expression of defense genes to prevent pathogen invasion [8]. As the second layer, ETI is mediated primarily by resistance (R) proteins, typically nucleotide-binding leucine-rich repeat (NLR) receptors, that recognize pathogen effectors either directly or through guardee/decoys. ETI primarily induces a hypersensitive response, characterized by programmed cell death, plant hormone synthesis, and defense-related gene expression [9]. Previous studies have shown that salicylic acid (SA), jasmonic acid (JA), and ethylene (ET) signaling pathways contribute to pathogen resistance; however, the SA pathway may antagonize or synergize with the JA and ET pathways [10,11,12,13]. SA functions as an endogenous signal molecule in the systemic acquired resistance (SAR) signaling pathway and has been identified in *tobacco*, *cucumber*, and *Arabidopsis* [14]. Nucleotide-binding site–leucine-rich repeat proteins play a critical role in ETI [15]. The central component of signal transduction in both PTI and ETI is the mitogen-activated protein kinase (MAPK) cascade, which activates immune regulation genes, such as *PR1*, and transcription factors, including the WRKY family [16,17]. The MAPK cascade includes three enzyme classes, MAPK, MAPK kinase (MAPKK), and MAPKK kinase (MAPKKK), which sequentially phosphorylate downstream substrates [18]. In *Arabidopsis*, WRKY22 and WRKY29 are transcriptionally induced by MPK3 and MPK6, conferring resistance to bacterial and fungal pathogens [19]. WRKYs are plant-specific transcription factors (TFs), involved in plant growth, development, secondary metabolite biosynthesis, and responses to biotic and abiotic stress [20]. Previous studies have reported that PagWRKY18 participates in immune responses and maintains intracellular homeostasis through PTI–ETI crosstalk [21]. WRKY TFs can activate or repress downstream genes by binding to W-box [TTGAC(C/T)] elements in promoter regions [22]. However, the role of WRKY TFs in directly regulating pathogenesis-related (PR) genes that trigger programmed cell death and modulate the reactive oxygen species balance in Chinese cabbage remains unclear. Overall, profiling transcript changes during both PTI and ETI is essential for understanding plant responses to clubroot pathogens.

In this study, we performed a global transcriptome analysis of Chinese cabbage treated with *P. brassicae* to identify the key genes involved in clubroot disease in cruciferous crops. Functional enrichment analysis of differentially expressed genes (DEGs) was used to investigate resistance-related pathways at different infection stages. Additionally, weighted gene coexpression network analysis (WGCNA) was performed to identify key genes related to resistance pathways within each module. Overall, the findings of this study offer insights into the interaction mechanism between Chinese cabbage and *P. brassicae* during infection.

## 2. Results

### 2.1. Phenotypic Observations and Transcriptome Overview

**Figure 1:**
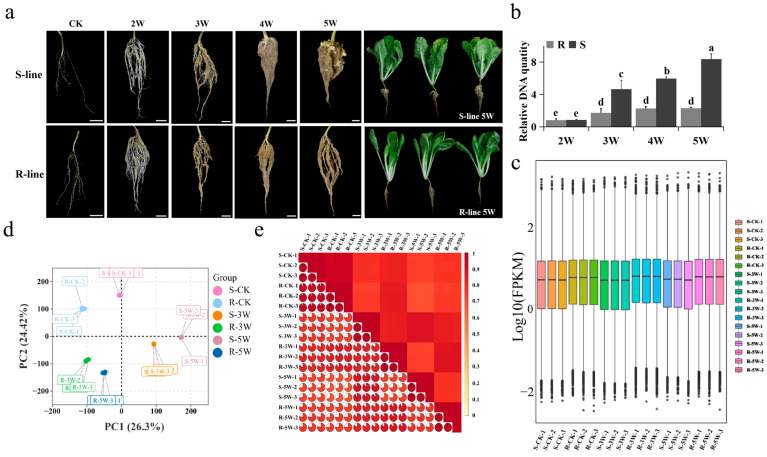
Phenotypic observations and transcriptome overview of Chinese cabbage upon *P. brassicae* inoculation. (**a**) Clubroot symptoms in the roots of S- and R-lines at 0, 2, 3, 4 and 5 weeks (marked as CK, 2W, 3W, 4W and 5W) after *P. brassicae* inoculation; (**b**) The relative content of *P. brassicae* was detected at 2, 3, 4 and 5 weeks of roots of R- and S-lines. The values represent the average of three biological replicates (*n* = 3). Error bars represent the mean ± standard deviation (SD) of three independent experiments. Distinct letters indicate significant differences at *p* < 0.05; (**c**) Expression levels of all samples; (**d**) Pearson correlation analysis; (**e**) Principal component analysis.

No visible clubroot symptoms were observed in either the resistant (R-line) “JJ S5-1” or susceptible line (S-line) “SYY1-10” until 21 days after *P. brassicae* inoculation. At 35 days, R-line roots remained free of gall formation, whereas irregular swellings appeared on both the lateral and main roots of S-line plants, indicating a successful infection suitable for further study (Figure 1a). Phenotypic differences between the R-line and S-line were analyzed and presented in Figure 1b. To explore resistance mechanisms in the two lines, 18 RNA libraries were constructed from RNA samples across three time points—control, mid-infection, and late infection—each with three replicates from the R- and S-lines. Samples were designated as CK, 3W, and 5W, corresponding to 0, 21, and 35 days after treatment, respectively, with distilled water or *P. brassicae*.

RNA sequencing (RNA-seq) generated approximately 134.41 Gb of clean data (~7 Gb per sample) from 18 libraries. Clean read rates exceeded 97% for all samples, with Q30 scores above 93% (Table S1). Between 86.28% and 92.38% of high-quality reads were mapped to version 3.5 of the Chinese cabbage genome, with more than 84% uniquely mapped reads (Table S1), confirming the accuracy and quality of the data for further analysis.

Gene expression levels were higher in the R-line than in the S-line (Figure 1c). Principal component analysis plot of gene expression levels indicated that the sample groups were distinct from each other, and the replicate samples within each group clustered closely together (Figure 1d). Pearson correlation analysis using fragments per kilobase of transcript per million mapped reads (FPKM) values was performed for the 18 samples, revealing a strong correlation among the three biological replicates in each group. The Pearson’s correlation coefficients for the replicates exceeded 0.99, confirming the accuracy and reliability of the inoculation treatment (Figure 1e).

### 2.2. DEGs in the R- and S-Lines at Different Time Points

To eliminate background differences between resistant and susceptible materials, relative transcriptional changes were analyzed over time. Differentially expressed genes (DEGs) at 3 and 5 weeks post-inoculation (WPI) were identified by comparing gene expression levels between 3W and CK and between 5W and CK within each line.

**Figure 2:**
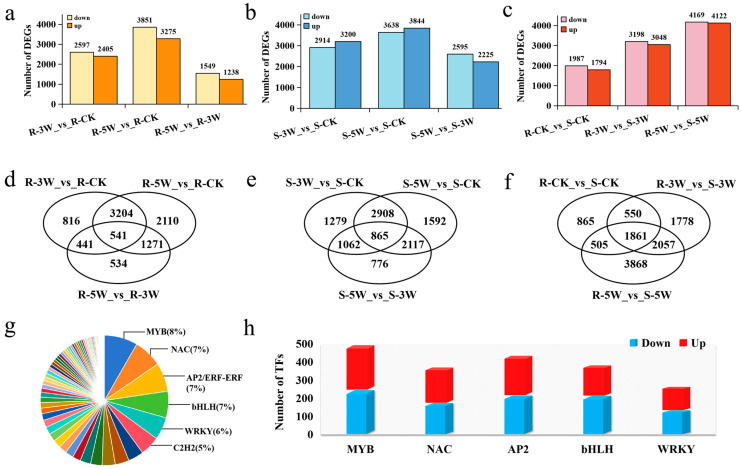
DEGs analysis of R- and S-line in response to *P. brassica* inoculation. (**a**) DEGs at 3W and 5W after *P. brassicae* inoculation in R-line; (**b**) DEGs at 3W and 5W after *P. brassicae* inoculation in S-line; (**c**) Comparison of DEGs at 3W and 5W after *P. brassicae* inoculation in R- and S-line; (**d**) Venn diagram of DEGs among R-3W vs. R-CK, R-5W vs. R-CK and R-5W vs. R-3W; (**e**) Venn diagram of DEGs among S-3W vs. S-CK, S-5W vs. S-CK and S-5W vs. S-3W; (**f**) Venn diagram of DEGs among R-CK vs. S-CK, R-3W vs. S-3W and R-5W vs. S-5W; (**g**) Classification of differentially expressed transcription factors; (**h**) Number of upregulated and downregulated genes of MYB, NAC, AP2, bHLH and WRKY transcription factors.

DEGs between samples with and without *P. brassicae* inoculation were identified using the following criteria: |log2 fold change| ≥ 1 and false discovery rate (FDR) < 0.05, resulting in the identification of 16,481 DEGs (Table S2). At 5 WPI, the number of DEGs in the R-line was approximately 1.5-fold higher than that of 3 WPI, indicating a stronger transcriptional response in the resistant strain during the later stage of infection. Comparison of R- and S- lines at different time points revealed that the highest number of DEGs was observed at 5 WPI, indicating a more pronounced response to *P. brassicae* at this stage (Figure 2a–c). In addition, Venn diagrams generated for nine comparison groups at three time points after inoculation revealed that 541 DEGs were consistently expressed across two time points in the R-line, whereas 865 DEGs were identified in the S-line. Comparison between the R- and S-lines at various time points revealed 1861 common DEGs were detected (Figure 2d–f). Figure 2f shows a total of 3918 genes consistently expressed at different time points after inoculation, among which 2057 DEGs may be involved in the responses of Chinese cabbage to *P. brassicae* inoculation.

Among the DEGs, 1643 were TFs (Table S3). The MYB, NAC, AP2, bHLH, and WRKY families were most involved in the clubroot disease response, with 138, 118, 117, 109, and 92 DEGs, respectively, in these families (Figure 2g,h).

**Figure 3:**
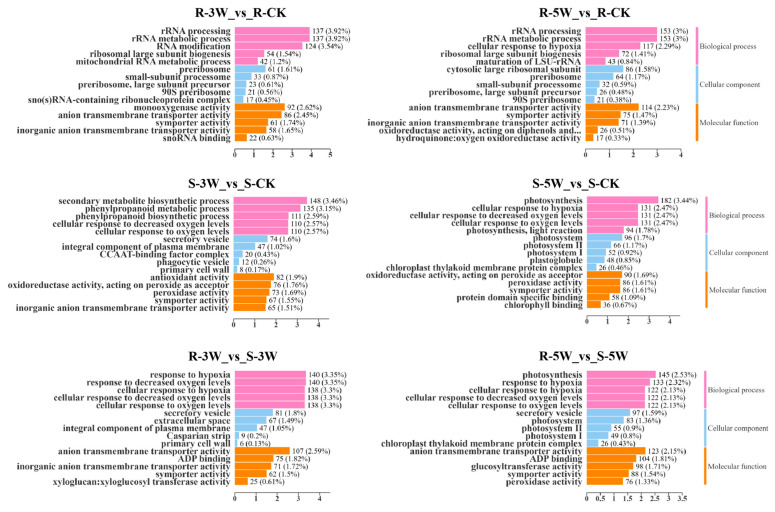
GO analysis of DEGs. The *Y*-axis represents GO terms grouped by functional classification: biological process (pink), molecular function (blue), cellular component (orange). The *X*-axis represents the percentage of DEGs enriched in each pathway relative to total annotated genes.

Gene Ontology (GO) enrichment analysis of R- and S-line DEGs classified their functions as biological processes, cellular components, and molecular functions. Top 5 of the three categories revealed that mitochondrial RNA modification and cellular response to extracellular stimulus were significantly enriched during mid-infection, whereas secondary metabolite biosynthesis was enriched during late infection (Figure 3). Detail GO enrichment analysis suggested that R-lines may modulate resistance phenotypes through alterations in protein synthesis efficiency and cellular metabolic homeostasis. Additionally, systemic acquired resistance (GO:0009627) was significantlly enriched in R-5W_vs_R-CK gruop (Table S4). In S-lines, the phenylpropanoid biosynthetic process (GO:0009699) and lignin metabolic process (GO:0009808) were significantly enriched at 3 WPI, suggesting that the host restricts pathogen spread via cell wall reinforcement. At later stage, enrichment shifted primarily to glucosinolate metabolic process (GO:0019760) and response to toxic substance (GO:0009636) (Table S4). Collectively, GO enrichment analysis demonstrated distinct resistance strategies: the R-line establishes immunity through mitochondrial functional reprogramming and systemic acquired resistance (SAR) activation, whereas the S-line employs phenylpropanoid-lignin pathway-mediated physical barriers.

**Figure 4:**
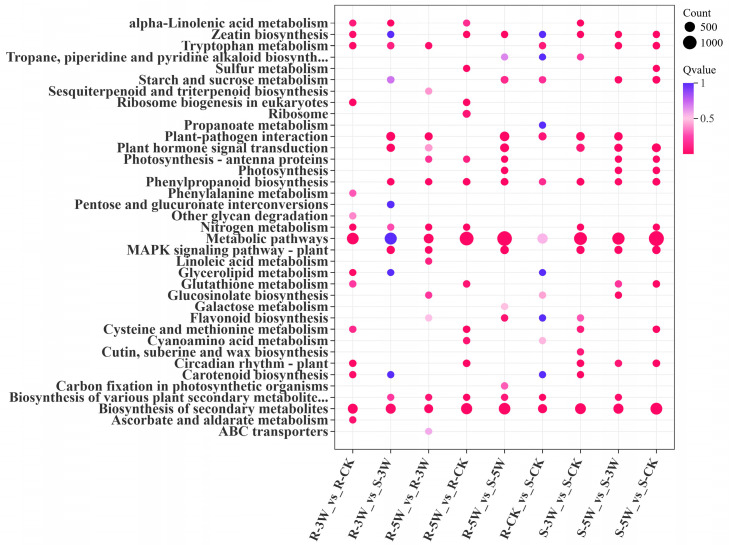
Top 15 terms of KEGG analysis of DEGs for each group. Bubble plot showing pathway enrichment analysis, with the *X*-axis indicating comparison groups, the *Y*-axis displaying enriched pathways; Dot size representing the number of enriched genes, and dot color reflecting the statistical significance of enrichment (FDR < 0.05).

To further explore DEG functions, the top 15 significantly enriched Kyoto Encyclopedia of Genes and Genomes (KEGG) pathways per group were identified (Figure 4). α-Linolenic acid metabolism (ko00592) was enriched between the R- and S-lines at 3 WPI. Plant hormone signal transduction (ko04075) and MAPK signaling pathway (plant) (ko04016) were consistently enriched between lines after inoculation. In the R-line, DEGs in 3W and CK were uniquely enriched in phenylalanine metabolism (ko00360). Metabolic pathways (ko01100) and secondary metabolite biosynthesis (ko01110) were significantly enriched across all comparisons. In addition to metabolic pathways (ko01100), plant–pathogen interaction (ko04626) accounted for the largest number of DEGs, followed by plant hormone signal transduction (ko04075) and MAPK signaling pathway (plant) (ko04016) (Table S5). Among these pathways, genes encoding disease resistance proteins (e.g., PR1 and LRR receptor-like serine/threonine-protein kinases) were predominantly upregulated in the resistant (R) line. Transcriptional analysis revealed that WRKY transcription factors were predominantly enriched in the pathway of MAPK signaling pathway (plant) and plant-pathogen interaction, where they differentially modulated disease resistance between resistant and susceptible genotypes.

### 2.3. DEG Coexpression Clusters and Functional Analysis

**Figure 5:**
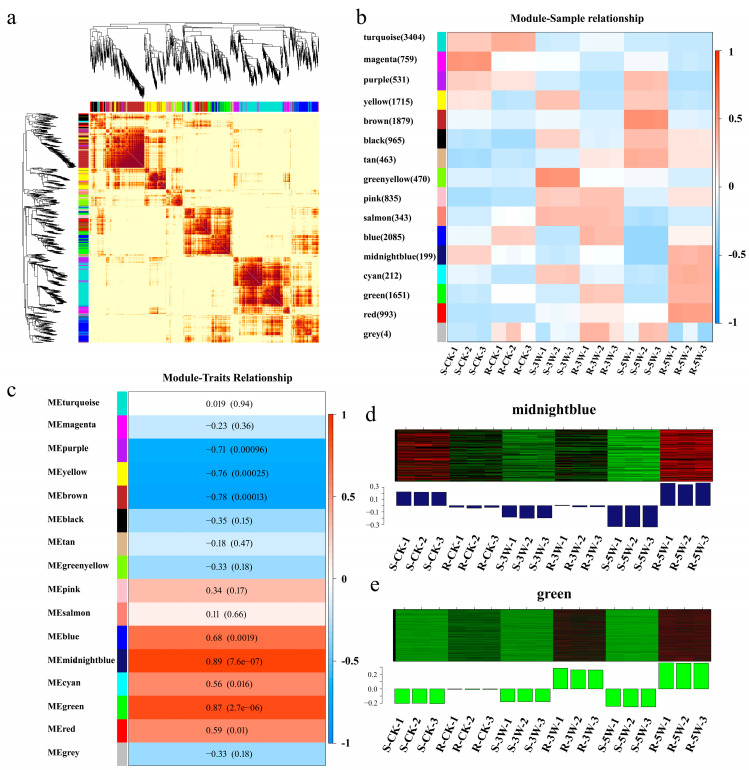
WGCNA of DEGs in R- and S-line at 0, 3, 5 WPI. (**a**) Hierarchical clustering tree generated by WGCNA; (**b**) Module-sample group association analysis. Each row represents a module (labeled with the same color as in (**a**)), and each column corresponds to a sample group. The color intensity in each cell reflects the correlation coefficient between the module and the sample group; (**c**) Correlation analysis between modules and *P. brassicae* infected samples of resistant (R) and susceptible (S) lines. The value in each cell indicates the correlation coefficient between the module genes and infected samples, with the corresponding *p*-value displayed beside; (**d**) Expression trends of DEGs in midnight blue module; (**e**) Expression trends of DEGs in green module.

WGCNA was performed on 16,481 DEGs after inoculation to better understand gene expression across developmental stages in both planting strains and identify genes highly associated with *P. brassicae* response. These DEGs were grouped into 16 distinct modules (indicated by color), with each tree branch representing a module and each leaf representing a gene (Figure 5a). Each module included genes with closely related expression patterns during *P. brassicae* infection. The magenta, brown, and green-yellow modules correlated with the R- or S-line (*p* > 0.5; Figure 5b). Genes in the purple, yellow, and brown modules were likely involved in the negative regulation of plant disease response (*p* > 0.7; Figure 5c). Notably, the midnight blue and green modules were substantially involved in disease response with positive regulation (*p* > 0.8; Figure 5d,e).

KEGG pathway enrichment analysis was performed on these key modules. Genes in the midnight blue module were enriched in plant–pathogen interactions (ko04626), ABC transporters (ko02010), zeatin biosynthesis (ko00908), flavone and flavonol biosynthesis (ko00944), secondary metabolite biosynthesis (ko01110), and MAPK signaling pathway (plant) (ko04016). Genes in the green module were enriched in plant–pathogen interactions (ko04626); MAPK signaling pathway (plant) (ko04016); plant hormone signal transduction (ko04075); cutin, suberine, and wax biosynthesis (ko00073); tryptophan metabolism (ko00380); inositol phosphate metabolism (ko00562); glycosphingolipid biosynthesis–lacto and neolacto series (ko00601); and glycerolipid metabolism (ko00561) (Table 1).

### 2.4. Identification of Key Genes Responding to P. brassicae Inoculation

The midnight blue and green modules contained 1850 DEGs between the R- and S-lines at the three time points (Table S6). KEGG pathway enrichment analysis of these DEGs revealed involvement in plant–pathogen interactions (ko04626); MAPK signaling pathway (plant) (ko04016); plant hormone signal transduction (ko04075); and cutin, suberine, and wax biosynthesis (ko00073) (Figure S1).

**Figure 6:**
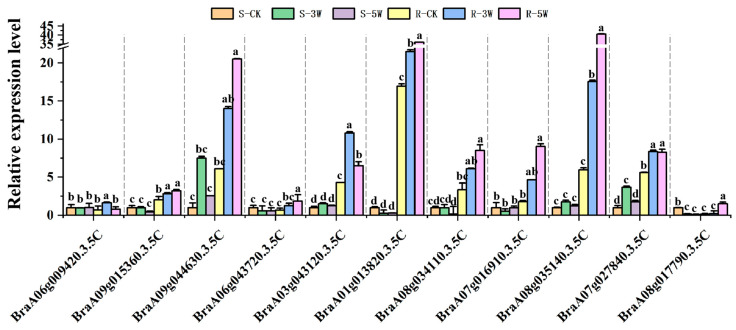
qRT-PCR analysis of DEGs. The values represent the average of three biological replicates. Error bars represent the mean ± standard deviation (SD) of three independent experiments. Distinct letters indicate significant differences at *p* < 0.05.

Among the differentially expressed genes (DEGs) specifically identified in R-3W_vs._S-3W and R-5W_vs._S-5W (but not in R-CK_vs._S-CK), we identified 11 DEGs with vital roles in the defense process of *P. brassica*, including disease resistance proteins (e.g., *BraA06g009420.3.5C*, *BraA09g015360.3.5C*, *BraA09g044630.3.5C*, and *BraA06g043720.3.5C*), PR proteins (e.g., *BraA03g043120.3.5C*), senescence-induced receptor-like serine/threonine-protein kinases (e.g., *BraA08g034110.3.5C*, *BraA07g016910.3.5C*, and *BraA08g035140.3.5C*), and serine/threonine-protein kinase PBS1 (e.g., *BraA01g013820.3.5C*), transcription factor TGA (*BraA07g027840.3.5C*) and WRKY18 (*BraA08g017790.3.5C*) (Figure 6).

Almost all genes (expect *BraA06g009420.3.5C*) were significantlly upregulated in the R-line after 5-week of *P. brassicae* inoculation compared with CK group. Among these, *BraA09g044630.3.5C*, *BraA01g013820.3.5C*, and *BraA08g035140.3.5C* expression levels were significantly upregulated by 20–40-fold in the R-line 5W group compared with the S-line CK group. SA-related signals associated with disease resistance were upregulated in the R-line, qRT-PCR showed that the expression level of *BraA07g027840.3.5C* was gradually increased after *P. brassica* inoculation. TFs are crucial in regulating plant development and defense, acting as both positive and negative regulators. Many WRKY genes are rapidly induced following *flg22* treatment in *Arabidopsis thaliana* [23]. Combining WGCNA, DEG analysis, and gene annotation, *BraA08g017790.3.5C* expression was found to be suppressed in the S-line after *P. brassicae* inoculation, whereas the R-line showed the opposite trend was observed in the R-line. WRKY18 was also significantly differentially expressed in a direct R-line versus S-line comparison (Figure 6 and Table S7).

**Figure 7:**
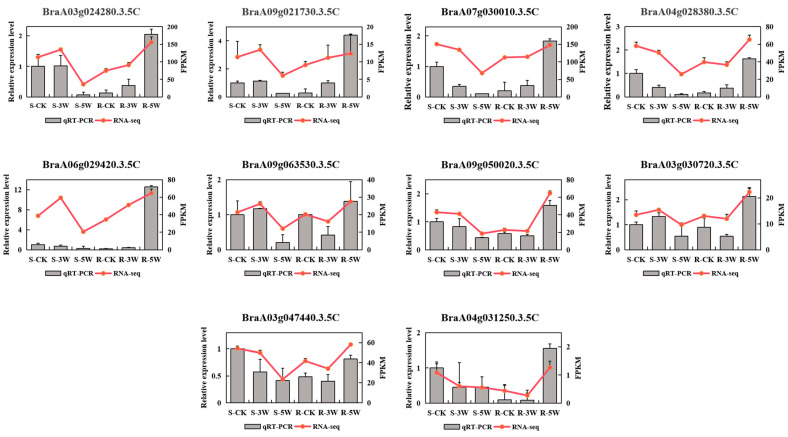
qRT-PCR expression validation for 10 selected genes. The gene names corresponding to each histogram are displayed above their respective bars. Left *Y*-axis represents qRT-PCR values, and right *Y*-axis represents FPKM values. The values represent the means ± SDs (*n* = 3) of three biological replicates.

Based on the expression profiles and coexpression analysis, 10 defense genes related to *P. brassica* infection were random selected for real-time qPCR (qRT-PCR) validation, including 3 WRKY, 1 CML, 1 MYC2, 2 PBS1, 1 RPS4, 1 calcium-dependent protein kinase and 1 somatic embryogenesis receptor kinase 4 (Supplementary Table S7). In S-lines, transcript levels of defense-related genes *BraA03g024280.3.5C* (WRKY22), *BraA09g021730.3.5C* (PBS1), *BraA06g029420.3.5C* (WRKY25), *BraA03g030720.3.5C* (CML), and *BraA09g063530.3.5C* (somatic embryogenesis receptor kinase 4) exhibited an initial increase followed by decline, peaking at 3WPI. In contrast, R-lines showed opposing expression patterns for *BraA03g030720.3.5C* and *BraA09g063530.3.5C*, while displaying sustained upregulation of *BraA03g024280.3.5C, BraA09g021730.3.5C*, and *BraA06g029420.3.5C*. In S-lines, transcript levels of defense-related genes *BraA09g050020.3.5C* (calcium-dependent protein kinase), *BraA07g030010.3.5C* (RPS4), *BraA04g028380.3.5C* (WRKY33), *BraA03g047440.3.5C* (MYC2), and *BraA04g031250.3.5C* (PBS1) were downregulated. Conversely, R-lines showed an initial decrease followed by subsequent upregulation of these genes. The qRT-PCR results closely matched RNA-seq data and revealed significant differential expression among samples, confirming the reliability of transcriptomic profiling (Figure 7).

**Figure 8:**
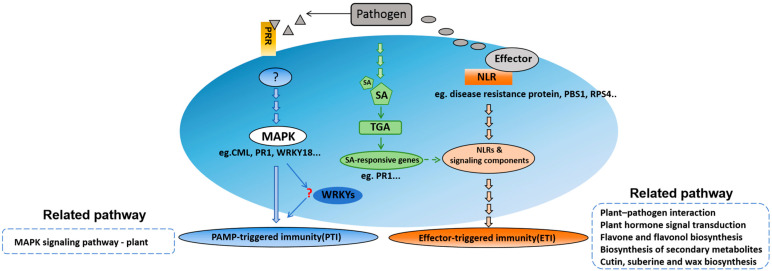
Speculation on the clubroot disease resistance mechanism of *P. brassicae* infection in Chinese cabbage.

A network diagram (Figure 8) was created to provide a clearer understanding of the defense mechanism induced by *P. brassicae*. Plants initiate primary defense responses via MAPK signaling, integrate hormone signaling, and employ resistance proteins to recognize pathogen effectors, triggering ETI and enhancing disease resistance specificity. These genes may be critical contributors to clubroot resistance in Chinese cabbage.

## 3. Discussion

Clubroot is a globally prevalent disease that severely threatens the growth, quality, and productivity of cruciferous crops [24]. The causal agent *P. brassicae* is a soilborne, obligate biotrophic protist with a complex life cycle, making it challenging to elucidate the molecular mechanisms underlying plant–pathogen interactions [25]. To obtain molecular insights into the invasion and defense strategies involved in *P. brassicae*-*Brassica* interactions, high-quality transcriptome sequencing was performed on resistant and susceptible varieties. The results expand current transcriptomic dataset resources and offer valuable insights into clubroot resistance mechanisms in Chinese cabbage.

Our findings revealed that *P. brassicae* infection progressively altered gene expression in host roots, with the highest number of DEGs identified at 5 WPI in the R- and S-line 5W groups. This pattern is consistent with the findings of previous studies, such as those reporting peak DEG numbers in W4Pb-infected Chinese cabbage [26]. These results indicate that *P. brassicae* induces significant transcriptomic changes in Chinese cabbage over time, particularly during the later stages of infection. Furthermore, the differential abundance of DEGs between R-line and S-line genotypes suggested enhanced sensitivity of the S-line to *P. brassicae* infection. Notably, the S-line exhibited significantly more DEGs than the R-line following pathogen challenge, consistent with its susceptible phenotype. The potential functional linkage between these DEGs and clubroot resistance requires further experimental validation assays.

GO enrichment analyses showed that both R- and S-lines had upregulated DEGs associated with stimulus–response pathways upon *P. brassicae* inoculation. Specifically, 64 DEGs in the R-line 5W group were implicated in SAR. This is consistent with previous transcriptome studies on *Albugo candida*-infected cultivars, which reported the activation of SAR-related genes and programmed cell death in resistant varieties [27]. In our KEGG pathway enrichment analysis, α-linolenic acid metabolism (ko00592) was notably activated in the R- and S-line 3W groups. This pathway is associated with cutin and wax biosynthesis during fungal infection, as shown in the *Prunus domestica-Monilinia fructigena* interaction [28]. Additionally, many DEGs were involved in phenylpropanoid biosynthesis, which is a mechanism of action under *P. brassicae* infection. Phenylpropanoids serve as the precursors of numerous disease-related secondary metabolites in plants, including flavonoids, isoflavones, and lignin [29,30]. The most enriched pathways in our study included those related to metabolism (ko01100), plant–pathogen interactions (ko04626), plant hormone signal transduction (ko04075), and MAPK signaling (plant) (ko04016). Previous studies have shown that genes related to secondary metabolites, disease resistance, and phytohormone signaling offer long-term defense against pathogens [31,32].

WGCNA revealed two key gene modules, midnight blue and green, that were highly correlated with clubroot resistance. Four KEGG pathways were significantly enriched within these modules: plant–pathogen interaction (ko04626); plant hormone signal transduction (ko04075); MAPK signaling pathway (plant) (ko04016); and cutin, suberine, and wax biosynthesis (ko00073) (Table 1). Overlap among genes involved in MAPK and hormone signaling indicates that disease resistance proteins, phytohormones, disease-related TFs, and cutin-related enzymes may contribute to clubroot resistance in Chinese cabbage.

Disease resistance genes and PR proteins play crucial roles in pathogen defense in plants [33,34]. In the present study, the expression of certain resistance-related DEGs and PR proteins was significantly altered after *P. brassicae* inoculation in our study (Figure 5). Pathogenesis-related genes, including *PR1*, *PR2*, and *PR4*, are consistently upregulated during *P. brassicae* infection in *B. napus* [35]. The PR1 protein (*BraA03g043120.3.5C*) was upregulated 7.2-fold during infection in the R-line compared with the S-line in 3W groups. Phytohormones, such as SA, JA, and ET, play crucial roles in plant immunity [36]. SA pathway activation typically suppresses JA signaling, and vice versa. However, under certain plant species or environmental conditions, these pathways may exhibit synergistic interactions. In *Arabidopsis*, NPR1 interacts with MYC2, and this interaction disrupts MYC2-MED25 complex formation, thereby suppressing JA-mediated transcriptional activation [37]. In rice, NPR1 competitively disrupts JAZ-MYC2 interactions, thereby releasing JA signaling [38]. Furthermore, the ethylene-responsive transcription factor EIN3 interacts with and suppresses MYC2 transcriptional activity [39,40]. These findings reveal the remarkable complexity and diversity of SA, JA and ET pathway crosstalk in plant immune regulation. Increased SA and JA signaling and accumulation have been observed in resistant rice mutants [41]. Additionally, SA content in host plants increases significantly during pathogen invasion, with SA-related genes activated through the SA biosynthesis pathway, thereby enhancing plant resistance to pathogens [42]. In the present study, significant changes in the expression levels of PR1 (*BraA03g043120.3.5C*) and TGA (*BraA07g027840.3.5C*), both components of the SA pathway, were detected between the R- and S-lines, indicating that SA-mediated defense plays a critical role in resisting *P. brassicae*. Previous studies have confirmed that PR1 is transcriptionally activated by TGA binding to its promoter, enhancing pathogen resistance [43,44]. Notably, the promoter region of *BraA03g043120.3.5C* lacks canonical TGA-binding cis-elements, suggesting that transcription factor TGA may interactions with other immune components.

In this study, the expression levels of disease resistance proteins (*BraA09g044630.3.5C*), PBS1 (*BraA01g013820.3.5C*) and senescence-induced receptor-like serine/threonine-protein kinases (*BraA08g035140.3.5C*) were significantly upregulated by 20–40-fold in the R-line 5W group compared with the S-line CK group. Research has demonstrated that PBS1 enhances plant immune responses by functioning as a decoy protein, safeguarding host proteins from degradation by pathogenic effector proteins. Furthermore, PBS1 contributes to pattern-triggered immunity (PTI) by phosphorylating key components of the MAPK signaling cascade, thereby amplifying defense responses against pathogens [45]. WRKY TFs, which are known regulators of plant immunity, were differentially expressed. WRKY18 (*BraA08g017790.3.5C*), a key gene in the midnight blue module, was downregulated in the S-line but upregulated in the R-line post-inoculation, indicating its potential role in inducing PTI during *P. brassicae* infection. WRKY18 binds to the W-box of SA biosynthesis (ICS1) and abscisic acid biosynthesis (NCED3 and NCED5) genes, contributing to NJ01-induced bacterial resistance [46]. Schön et al. [47] reported that WRKY18 and WRKY40 act as positive regulators of ETI in tomato plants. Previous studies have shown that the WRKY18-CHS3 axis is involved in the resistance of pear plants to black spot disease [48]. These results highlight the conserved role of WRKY TFs in resistance to plant diseases. Cutin and wax biosynthesis also function as inducible resistance components, forming as physical and chemical barriers against pathogen infection. Phenylalanine, a precursor for cutin and wax biosynthesis, is regulated by the JA and SA pathways [49]. In the present study, the gene *BraA09g025620.3.5C*, annotated as a stemmadenine O-acetyltransferase, was identified in the cutin, suberine, and wax biosynthesis pathways. This gene is a promising target for future research to enhance stress resistance in crops.

Overall, these results indicate that the identified resistance-related DEGs, particularly those associated with SA signaling, PBS1, PR proteins, WRKY TFs, and cutin biosynthesis, play key roles in Chinese cabbage’s defense against *P. brassicae*. Further functional validation of these genes will deepen our understanding of host–pathogen interactions between Chinese cabbage and *P. brassicae* and may contribute to the breeding of clubroot-resistant cultivars.

## 4. Materials and Methods

### 4.1. Plant Materials and P. brassicae Inoculation

Two commercial Chinese cabbage (*Brassica rapa* ssp. *pekinensis*) cultivars, ‘Jinjin’ and ‘Shuishiying 91-12’, were self-pollinated for multiple generations to generate two advanced inbred lines, ‘JJ-S5-1’ (derived from ‘Jinjin’) and ‘SYY-10’ (derived from ‘Shuishiying 91-12’). Two inbred lines of Chinese cabbage (JJ S5-1: resistant, R-line, *B. rapa*, 2n = 2x = 20; SYY-10: susceptible, S-line, *B. rapa*, 2n = 2x = 20), both of which were previously evaluated for clubroot resistance, were used in this study. Both lines were obtained from the Institute of Vegetable Research, Liaoning Academy of Agricultural Sciences, China. The *P. brassicae* strain—classified as Pb4 under the SCD system—originated from Xinmin, Liaoning Province, China [50] and was propagated on susceptible Chinese cabbage lines. Fresh galls were stored at −20 °C for future use. All samples were stored at the Institute of Vegetable Research.

Seeds from both lines were germinated on moist filter paper in Petri dishes under controlled conditions (22 °C and a 16/8-h light/dark photoperiod) for 24 h before transplanting into 50-well plastic trays. A *P. brassicae* spore suspension was diluted to 1 × 10^7^ spores/mL and applied to the stem base of 21-day-old Chinese cabbage plants [51]. Root samples were collected at three time points: control (treatment with sterile water, 0 days after inoculation, CK), mid-infection (treatment with *P. brassicae*, 21 days after inoculation, 3W), and late infection (treatment with *P. brassicae*, 35 days after inoculation, 5W).

### 4.2. RNA Extraction, Library Preparation and Transcriptome Sequencing

Total RNA was extracted from 18 samples using ethanol precipitation and the CTAB-PBIOZOL method. RNA was dissolved in 50 µL of DEPC-treated water, and its quality and quantity were assessed using a Qubit fluorometer and a Qsep400 high-throughput biofragment analyzer. cDNA libraries were generated and sequenced on the Illumina Novaseq 6000 platform.

Raw reads were filtered using fastp to remove adapters and low-quality sequences. Reads containing > 10% ambiguous bases or >50% low-quality bases (Q ≤ 20) were discarded. HISAT was used to construct an index, and clean reads were aligned to the *B. rapa* reference genome (downloaded with its annotation files from http://39.100.233.196:82/download_genome/Brassica_Genome_data/Brara_Chiifu_V3.5) (accessed on 8 October 2023).

### 4.3. DEG Expression Analysis and Functional Annotation

Gene expression was quantified using featureCounts, and FPKM values for each gene were calculated based on gene length. Differential expression analysis between groups was performed using DESeq2, with Benjamini–Hochberg correction applied to *p*-values to control for FDR. DEGs were defined as genes with FDR < 0.05 and | log2 fold change| ≥ 1 in the comparative analysis. The FPKM values for the DEGs are listed in Supplementary Table S2.

Functional annotation of DEGs was performed using the NCBI nonredundant protein database, Cluster of Orthologous Groups database, GO database (http://geneontology.org/) (accessed on 30 October 2023) [52], and KEGG database (https://www.genome.jp/kegg) (accessed on 30 October 2023) [53]. Enrichment analysis was performed based on the hypergeometric test, with pathway-based hypergeometric distribution testing used for KEGG enrichment and GO term–based analysis used for GO enrichment. *p* < 0.05 indicated significantly enriched pathways and terms. The data background comprised genes from the whole genome.

### 4.4. WGCNA

WGCNA (version 1.71) in R was used to construct weighted gene coexpression networks. Modules with similar expression profiles were merged at a cut-height of 0.25.

### 4.5. Quantification of Gene Expression and RNA-Seq Data Evaluation

First-strand cDNA was synthesized using the PrimeScript™ RT Reagent Kit with gDNA Eraser (TaKaRa, Beijing, China) according to the manufacturer’s instructions. Primers were designed using Primer5.0 and synthesized by Synbio Tech (Suzhou, China). qRT-PCR was conducted using TB Green^®^ Premix Ex Taq™ (TaKaRa, Beijing, China) on a BIORAD CFX96 Real-Time PCR System, with 20-µL reaction systems. Each gene was analyzed in triplicate, and the relative expression was calculated using the 2^−∆∆Ct^ method [54]. Gene-specific primers are listed in Supplementary Table S8.

### 4.6. Statistical Analysis

Statistical analyses were performed using SPSS.22. Student’s *t*-test and one-way analysis of variance were used to evaluate group differences, followed by Duncan’s multiple range test. Statistical significance was defined as *p* < 0.05.

## 5. Conclusions

In this study, we analyzed the gene expression profiles in the resistant “JJ S5-1” and susceptible “SYY10-1” Chinese cabbage lines at various different stages of *P. brassicae* infection. We observed a progressive increase in the number of DEGs over time, with the highest number detected in the late infection stage. Transcriptomic and WGCNA analyses identified key resistance-related genes and pathways, including those involved in hormone signaling, MAPK signaling, and cutin biosynthesis. Notably, WRKY18 and PR1 were identified as candidate genes associated with resistance mechanisms. These findings provide valuable insights into the molecular basis of clubroot resistance in Chinese cabbage and provide a foundation for functional validation and breeding strategies targeting enhanced resistance enhancement.

## Figures and Tables

**Table 1:** KEGG enrichment analysis of modules that were significantly associated with trait in WGCNA analysis.

Module	KEGG_ID	Description	*p*-Value
Midnight blue	ko04626	Plant–pathogen interaction	5.36166 × 10^−5^
	ko02010	ABC transporters	0.001097744
	ko00908	Zeatin biosynthesis	0.011942757
	ko00944	Flavone and flavonol biosynthesis	0.03415298
	ko01110	Biosynthesis of secondary metabolites	0.034815157
	ko04016	MAPK signaling pathway–plant	0.045562111
green	ko04626	Plant-pathogen interaction	5.90315 × 10^−14^
	ko04016	MAPK signaling pathway-plant	4.13077 × 10^−5^
	ko04075	Plant hormone signal transduction	7.81607 × 10^−5^
	ko00073	Cutin, suberine and wax biosynthesis	0.012763979
	ko00380	Tryptophan metabolism	0.024950856
	ko00562	Inositol phosphate metabolism	0.029331177
	ko00601	Glycosphingolipid biosynthesis–lacto and neolacto series	0.042280796
	ko00561	Glycerolipid metabolism	0.049712822

## Data Availability

The raw sequence data reported in this paper have been deposited in the National Center for Biotechnology Information (NCBI) Sequence Read Archive (SRA) data under accession number PRJNA1251718 (https://www.ncbi.nlm.nih.gov/bioproject/PRJNA1251718 (accessed on 16 April 2025)).

## Supplementary Materials

The following supporting information can be downloaded at: https://www.mdpi.com/article/10.3390/plants14142105/s1, Figure S1: KEGG enrichment of DEGs in midnightblue and green; Table S1: Summarized data for sequenced samples; Table S2: Overview of DEGs; Table S3: Classification of transcription factors; Table S4: GO enrichment; Table S5: KEGG enrichment; Table S6: DEGs in module of midnight blue and green; Table S7: Sequence of selected genes; Table S8: Primers used in this study.
