# Supplementary figures and images for: Transcriptome and Coexpression Network Analyses Provide Insights into the Resistance of Chinese Cabbage During Different Stages of Plasmodiophora brassicae Infection
Source: Plants (Basel). 2025 Jul 8;14(14):2105. doi: 10.3390/plants14142105 (PMC12300438; doi:10.3390/plants14142105)

## midnightblue module

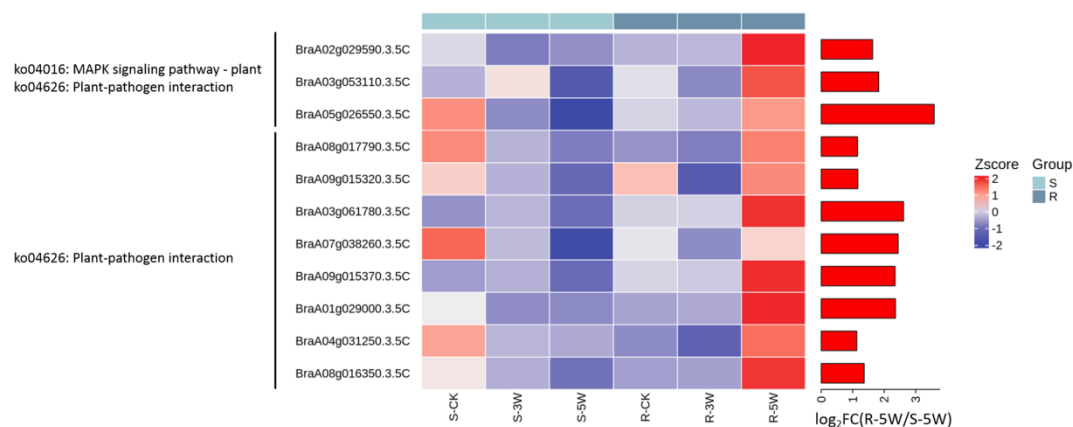

## green module

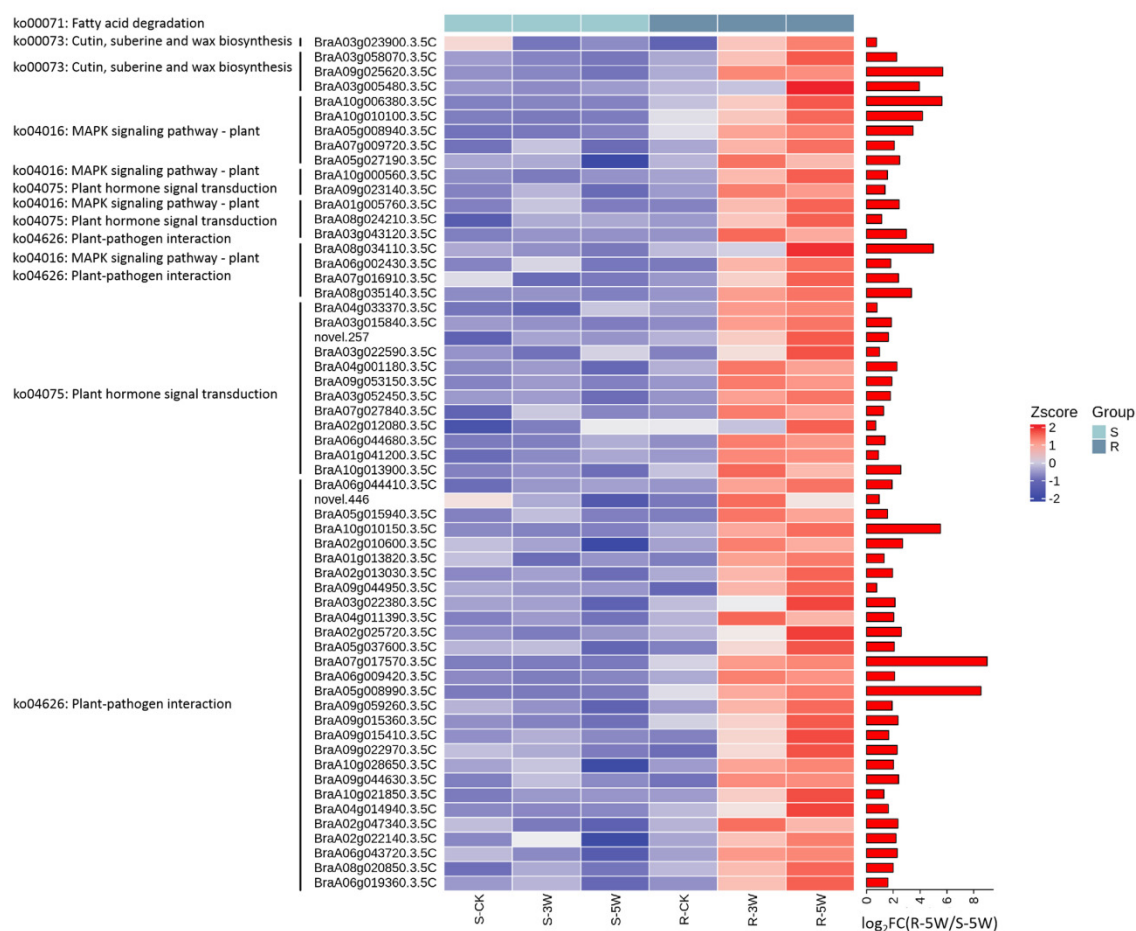

FigureS1. KEGG enrichment of DEGs in midnightblue and green

Supplement: Supplementary file 1 [file plants-14-02105-s001.zip › Supplementary Figures.pdf]
